# One Single Molecule as a Multifunctional Fluorescent Probe for Ratiometric Sensing of Fe^3+^, Cr^3+^ and Colorimetric Sensing of Cu^2+^

**DOI:** 10.3390/s150100049

**Published:** 2014-12-23

**Authors:** Yanqiu Yang, Kangkang Yu, Liang Yang, Jun Liu, Kun Li, Shunzhong Luo

**Affiliations:** 1 Institute of Nuclear Physics &Chemistry, China Academy of Engineering Physics (CAEP), Mianyang 621900, China; E-Mails: yan.qiu.yang@163.com (Y.Y.); leonesta@163.com (L.Y.); ljkxch@163.com (J.L.); 2 College of Chemistry, Sichuan University, Chengdu 610064, China; E-Mail: kangkangyu_1991@163.com

**Keywords:** rhodamine, multifunctional probe, click chemistry

## Abstract

The reagent **Rh-C**, incorporating a rhodamine moiety and a coumarin backbone and prepared *via* click chemistry, was developed as the first single molecule for detecting Cu^2+^, Fe^3+^ and Cr^3+^. Its response to Cu^2+^ in different solutions is visible to the naked eye and it exhibits a ratiometric fluorescence response to Fe^3+^ in methanol and Cr^3+^ in acetonitrile.

## 1. Introduction

Metal ions play very important roles in living system and have significant impacts on human health. Cu^2+^, Fe^3+^ and Cr^3+^ are indispensable nutrients for life, and both their deficiency and excesses are connected to various disorders [1–3]. For instance, during the uptake, storage and trafficking of Cu^2+^, a deficiency is proposed to be associated with severe diseases such as Menkes syndrome, Wilson's disease and Alzheimer's disease [4–6]. Fe^3+^ is one of the most essential metals in oxygen transport and electron transport [2,7,8]. Fe^3+^deficiency in our bodies will lead to low oxygen delivery, which could result in low blood pressure, anemia and decreased immunity [9,10]. On the contrary, excess Fe^3+^ can result in the formation of reactive oxygen species (ROS), which have damaging effects on lipids, nucleic acids, and proteins [11,12]. A normal person needs to absorb 25–35 mg of Cr^3+^ per day to improve the metabolism of glucose and lipids [13], while excessive intake of Cr^3+^ has an adverse effect on cellular structure [14,15]. In consideration of the necessity and toxicity of Cu^2+^, Fe^3+^ and Cr^3+^, the design of new probes for exact and convenient detection of Cu^2+^, Fe^3+^ and Cr^3+^ in environmental and biological samples is of much interest.

In recent years, because of the inexpensiveness, high sensitivity and simplicity of fluorescence analysis [16], fluorescent indicators have attracted widespread attention, and a rapidly increasing number of metal-responsive fluorescent sensors have been studied [17–19]. Although chemists have developed a great deal of fluorescent probes for Cu^2+^ [20–23], Fe^3+^ [24] and Cr^3+^ [25–30], respectively, probes which can indicate Cu^2+^, Fe^3+^ and Cr^3+^ at the same time *via* a single molecular species have not been reported. Herein, we intend to develop a fluorescent probe that could distinguish Cu^2+^, Fe^3+^ and Cr^3+^ under different conditions with one single molecule.

**Scheme 1.**
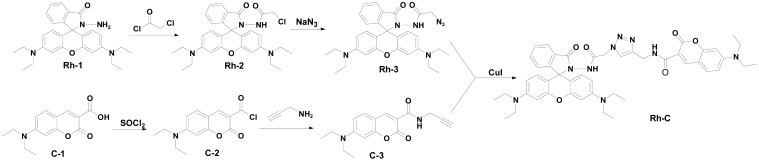
Synthesis of **Rh-C**.

According to previous studies of other researchers, rhodamine dyes are generally used as fluorescent probes because of their excellent photostability, photophysical properties and suitable water-solubility [31,32]. On the other hand, coumarin, as another well-known strongly fluorescent compound, is easy and convenient to synthesize in general [33]. To date, some FRET probes based on rhodamine and coumarin to achieve ratiometric fluorescent responses have been reported [34–36]. Inspired by these works, we sought to design a FRET ratiometric fluorescent probe **Rh-C** by connecting the rhodamine B and coumarin moieties with a 1,2,3-triazole linker (Scheme 1), in the hope that the introduction of the triazole system might provide an additional coordination site [37–39]. **Rh-C** and the intermediates were characterized by ^1^H-NMR, ^13^C-NMR, MS (see Supporting Information).

## 2. Results and Discussion

**Figure 1.**
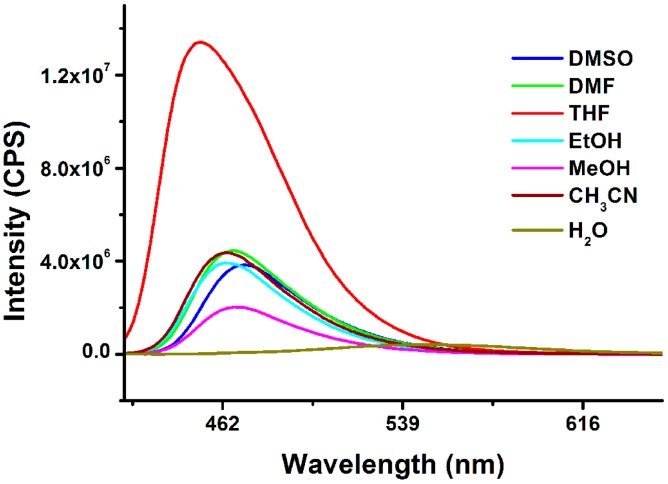
The fluorescence emission spectra of **Rh-C** (5 μM) in different solvents. (λ_ex_ = 400 nm).

Firstly, the fluorescence emission profiles of **Rh-C** in different solvents (DMSO, DMF, THF, EtOH, MeOH, MeCN, H_2_O) were studied. As shown in Figure 1, the emission peak of **Rh-C** at ∼470 nm which represented the characteristic peak of coumarin was almost unchanged in different organic solvents. Meanwhile, no peak was found at ∼580 nm corresponding to the emission of rhodamine B, indicating that the spirolactam of rhodamine moiety remained closed [31,32]. Surprisingly, the fluorescence of **Rh-C** was fairly weak and the emission peak was obviously red-shifted in water, which might be due to the TICT property of the 7-diethylamino group of the coumarin moiety [40]. Thus, the properties of **Rh-C** were mainly studied in organic solutions.

**Figure 2.**
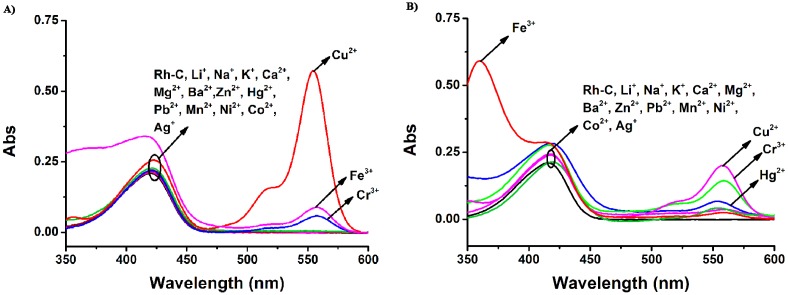

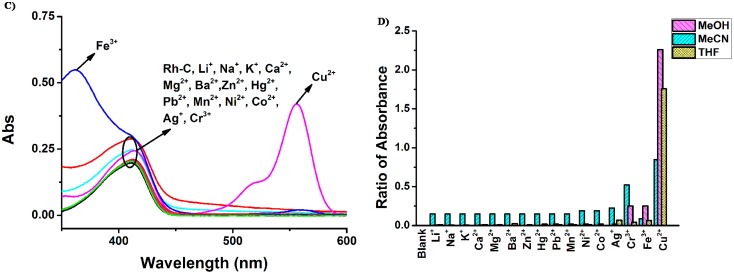
The UV-Vis absorption of **Rh-C** (5 μM) upon addition of different metal ions (100 μM). (**A**) in MeOH; (**B**) in MeCN; (**C**) in THF; (**D**) The ratiometric absorption of **Rh-C** (5 μM) upon addition of different metal ions (100 μM. MeOH: A_554_/A_420_, MeCN: A_558_/A_415_, THF: A_556_/A_410_).

Then, the cation selectivity of **Rh-C** in different solvents was investigated through UV-Vis absorption and fluorescence emission spectroscopy. As illustrated in Figure 2, the absorption peaks of **Rh-C** (5 μM) were located at around 415 nm in MeOH, MeCN and THF and the addition of 20 equiv of a range of physiologically and environmentally relevant metal ions (Li^+^, Na^+^, K^+^, Ca^2+^, Mg^2+^, Ba^2+^, Zn^2+^, Hg^2+^, Pb^2+^, Mn^2+^, Ni^2+^, Co^2+^ and Ag^+^) did not cause any significant changes in the absorption spectra. However, in MeOH solution, the addition of 20 equiv of Cr^3+^, Fe^3+^ and Cu^2+^ led to the appearance of a typical peak at 550 nm related to the opening of the spiro ring of rhodamine B (for Cr^3+^ 11-fold, for Fe^3+^ 17-fold, and for Cu^2+^ 114-fold), accompanied by a color change from light yellow to pink. In MeCN solution of **Rh-C**, the addition of Cu^2+^ and Cr^3+^ could cause a slight enhancement of the absorption at 558 nm, but only Cu^2+^ could induce a color change from light yellow to pink. In THF solution of **Rh**-**C**, only the addition of Cu^2+^ could cause a remarkable increase of absorption at ∼550 nm, implying that **Rh**-**C** exhibited an excellent selectivity towards Cu^2+^ in THF.

**Figure 3.**
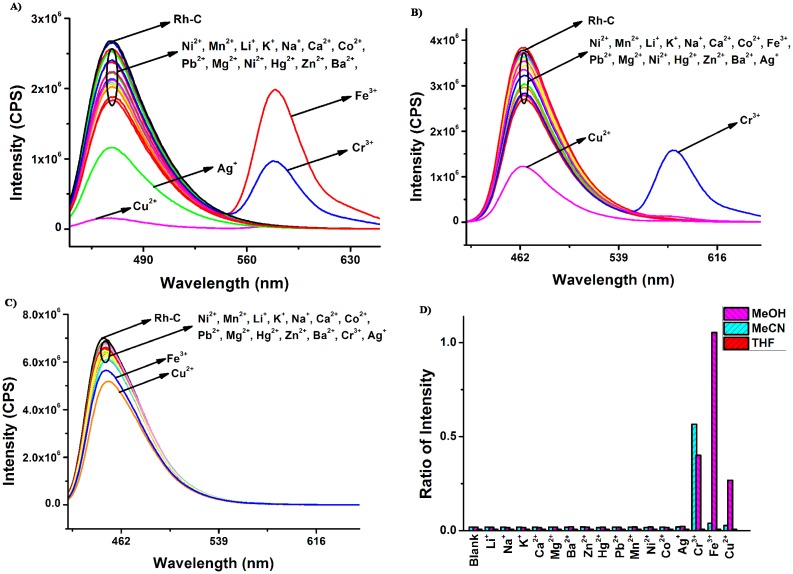
The fluorescence response of **Rh-C** (5 μM) upon addition of different metal ions (100 μM). (**A**) in MeOH (λ_ex_ = 420 nm); (**B**) in MeCN (λ_ex_ = 400 nm); (**C**) in THF (λ_ex_ = 400 nm); (**D**) The ratiometric absorption of **Rh-C** (5 μM) upon addition of different metal ions (100 μM. MeOH: *I*_579_*/I*_470_, MeCN: *I*_581_*/I*_463_, THF: *I*_580_*/*I_450_).

To sum up, **Rh**-**C** exhibited selective colorimetric response towards Cu^2+^ in different organic solution, particularly in THF. The fluorescence responses of **Rh**-**C** (5 μM) towards various metal ions are shown in Figure 3.

As envisioned, in MeOH, MeCN and THF, there were no significant changes in the fluorescence spectra after the addition of 20 equiv of Li^+^, Na^+^, K^+^, Ca^2+^, Mg^2+^, Ba^2+^, Zn^2+^, Hg^2+^, Pb^2+^, Mn^2+^, Ni^2+^ and Co^2+^, but surprisingly, the addition of Cu^2+^ did not result in the appearance of the typical emission peak of rhodamine B at ∼580 nm but rather led to more or less fluorescence quenching at ∼470 nm in all solvents, while the spiro ring opening of rhodamine B indeed occurred in the presence of Cu^2+^. We assumed that although the coordination between Cu^2+^ with **Rh-C** caused the spiro ring opening of rhodamine B, the fluorescence of the rhodamine B moiety as well as the coumarin was simultaneously quenched by Cu^2+^ due to the heavy metal ion effect [41]. In MeOH, upon addition of 20 eq. Cr^3+^ and Fe^3+^, an obvious fluorescence increment at 579 nm and a moderate fluorescence reduction at 470 nm was observed, indicating the generation of a FRET process between coumarin and rhodamine B. The emission intensity change ratios induced by Fe^3+^ and Cr^3+^ were 59-fold and 22-fold, respectively. In MeCN, only Cr^3+^ could induce the fluorescence increment of **Rh-C** at 581 nm, suggesting that **Rh-C** could selectively detect Cr^3+^ in MeCN *via* fluorimetry. In THF, the fluorescence of **Rh-C** showed almost no changes upon addition of the tested metal ions. In short, **Rh-C** exhibited a moderate selectivity toward Fe^3+^ in MeOH and an outstanding selectivity toward Cr^3+^ in MeCN through a FRET pathway.

**Figure 4.**
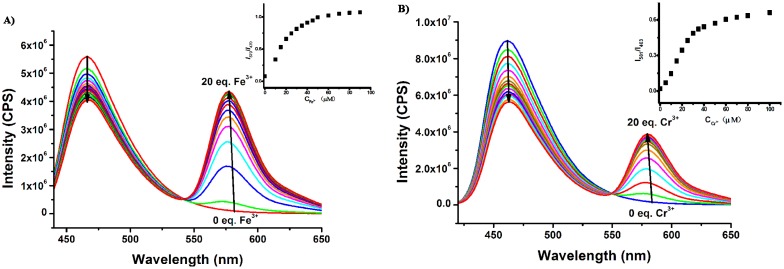
(**A**) The fluorescence response of **Rh-C** (5 μM) in MeOH solution upon addition of different amounts of Fe^3+^ (0–20 eq.); (**B**) The fluorescence response of **Rh-C** (5 μM) in MeCN solution upon addition of different amounts of Cr^3+^ (0–20 eq.). Inset: Ratiometric fluorescence intensity as a function of (A) Fe^3+^ [*I*_579_/*I*_470_], λ_ex_ = 420 nm; (B) Cr^3+^ [*I*_581_/*I*_463_], λ_ex_ = 400 nm.

Subsequently, the fluorescence titrations of **Rh-C** (5 μM) toward Fe^3+^ in MeOH and Cr^3+^ in MeCN were exploited, respectively. As depicted in Figure 4, with the addition of more of Fe^3+^ into the MeOH solution of **Rh-C**, the emission of **Rh-C** at 470 nm gradually decreased, and a new peak at 579 nm corresponding to the spiro ring opening of rhodamine B appeared (Figure 4A). A FRET process was speculated to emerge between the coumarin moiety and the rhodamine B fluorophore. The fluorescence intensity ratio (*I*_579_/*I*_470_) reached a plateau when 10 equiv Fe^3+^ was added, accompanied by a remarkable enhancement of the emission intensity ratio from 0.02 to 0.94. A similar phenomenon was observed upon addition of Cr^3+^ into the MeCN solution of **Rh-C**. The emission of **Rh-C** at 463 nm decreased gradually as the amount of Cr^3+^ in MeCN increased, accompanied with a fluorescence increase at 581 nm (Figure 4B). The fluorescence intensity ratio (*I*_581_/*I*_463_) became stable after 10 equiv of Cr^3+^ was added, with an enhancement of the emission intensity ratio from 0.02 to 0.68.

We believe that the different selectivity of **Rh-C** towards metals in different solvents (THF, MeOH, and MeCN) is closely related to the complexing abilities of the metals. All the ions (Cu^2+^, Fe^3+^, and Cr^3+^) complexed with **Rh-C** could induce ring-opening of the rhodamine moiety. In addition, the coumarin moiety, which shows an absorption band that peaks at∼415 nm, behaves as an energy donor for the FRET system, and the open form of the rhodamine moiety, which shows an absorption band that peaks at ∼550 nm, acts as an energy acceptor.

## 3. Experimental Section

### 3.1. General Information

^1^H-NMR and ^13^C-NMR spectra were measured on a Bruker AM400 NMR spectrometer (Fällanden, Switzerland). Proton chemical shifts of NMR spectra are given in ppm relative to the internal reference TMS (1H, 0.00 ppm). ESI-MS and HRMS spectral data were recorded on a Thermo Finnigan LCQ^DECA^ (San Jose, CA, USA) and a BrukerDaltonics Bio TOF mass spectrometer (Billerica, MA, USA), respectively. Fluorescence emission spectra were obtained using FluoroMax-4 Spectrofluorophotometer (HORIBA JobinYvon, Paris, France) at 298 K. Unless otherwise noted, materials were obtained from commercial suppliers and were used without further purification. All of the solvents were either HPLC or spectroscopic grade in the optical spectroscopic studies and they were dried according to the standard methods prior to use.

### 3.2. Synthesis of Various Compounds

**Rh-1**, **Rh-2**, **C-1** and **C-2** were synthesized according to the literature [42–44].

*Synthesis of 2-Azido-N-(3′,6′-bis(diethylamino)-3-oxospiro[isoindoline-1,9′-xanthen]-2-yl)acetamide* (**Rh-3**). Under nitrogen, **Rh-2** (650 mg, 1.2 mmol) was dissolved in DMF (15 mL), then sodium azide (234 mg, 3.6 mmol) was added to the solution very carefully. The mixture was stirred at 50 °C for 12 h. After the reaction, the solution extracted with CH_2_Cl_2_ (3 × 20 mL) and the combined organic layer was washed with brine (3 × 20 mL). After the solution was dried over anhydrous sodium sulfate, the solvent was removed under the reduced pressure and the residue was further purified by column chromatography to afford **Rh-3** (620 mg, 95.6%) as a white solid. ^1^H-NMR (400 MHz, DMSO) δ 9.91 (s, 1H), 7.84 (d, *J* = 6.9 Hz, 1H), 7.63–7.48 (m, 2H), 7.03 (d, *J* = 7.2 Hz, 1H), 6.51 (d, *J* = 9.3 Hz, 2H), 6.34 (d, *J* = 6.6 Hz, 4H), 3.99 (s, 2H), 3.34 (s, 8H), 1.09 (t, *J* = 6.8 Hz, 13H). MS: *m/z* [M+H]^+^ calcd. 540.2719 found, 540.2723.

*Synthesis of 7-(Diethylamino)-2-oxo-N-(prop-2-yn-1-yl)-2H-chromene-3-carboxamide* (**C-3**). Under nitrogen, triethylamine (1.4 mL, 10 mmol) was added to a solution of propargylamine (0.68 mL, 10 mmol) in anhydrous DCM and then a solution of C-2(1.4 g, 5 mmol) in anhydrous DCM was added dropwise to the solution with stirring. The organic layer was washed with water after stirred for 24 h. After the solution was dried over anhydrous sodium sulfate, the solvent was removed under the reduced pressure and the residue was further purified by column chromatography to afford **C-3** (1.3 g, 87.2%) as a yellow solid. ^1^H-NMR (400 MHz, CDCl_3_) δ 9.01 (s, 1H), 8.72 (s, 1H), 7.45 (d, *J* = 9.0 Hz, 1H), 6.67 (dd, *J* = 9.0, 2.5 Hz, 1H), 6.52 (d, *J* = 2.4 Hz, 1H), 4.25 (dd, *J* = 5.4, 2.5 Hz, 2H), 3.48 (q, *J* = 7.1 Hz, 4H), 2.26 (t, *J* = 2.5 Hz, 1H), 1.26 (t, *J* = 7.1 Hz, 6H).

*Synthesis of N-((1-(2-((3′,6′-bis(diethylamino)-3-oxospiro[isoindoline-1,9′-xanthen]-2-yl)amino)-2-oxoethyl)-1H-1,2,3-triazol-4-yl)methyl)-7-(diethylamino)-2-oxo-2H-chromene-3-carboxamide* (**Rh-C**). **Rh-3** (369 mg, 0.68 mmol), **C-3** (210 mg, 0.7 mmol) and CuI (285 mg, 1.5 mmol) were added in 20 mL THF-water (v:v, 1:1). The mixture was stirred at 50 °C for 24 h, and monitored by TLC. Then the solution was cooled down to room temperature and the solvent was removed under reduced pressure. After that, the solution was poured into distilled water and extracted with CH_2_Cl_2_ (3 × 30 mL). The combined extracts were dried over anhydrous sodium sulfate. The solvent was removed under the reduced pressure and the residue was further purified by column chromatography to afford **Rh-C** (204 mg, 35.8%) as an orange solid. ^1^H-NMR (400 MHz, DMSO) δ 10.12 (s, 1H), 9.02 (t, *J* = 5.5 Hz, 1H), 8.70 (s, 1H), 7.82 (d, *J* = 6.8 Hz, 1H), 7.73–7.67 (m, 2H), 7.61–7.48 (m, 2H), 7.01 (d, *J* = 7.3 Hz, 1H), 6.82 (dd, *J* = 9.1, 2.3 Hz, 1H), 6.63 (d, *J* = 2.0 Hz, 1H), 6.52 (d, *J* = 8.5 Hz, 2H), 6.34 (d, *J* = 9.2 Hz, 4H), 5.07 (s, 2H), 4.54 (d, *J* = 5.5 Hz, 2H), 3.49 (q, *J* = 6.9 Hz, 4H), 3.32 (d, *J* = 7.7 Hz, 8H), 1.15 (t, *J* = 7.0 Hz, 6H), 1.08 (t, *J* = 6.9 Hz, 12H). MS: m/z [M+H]^+^, calcd. 838.4041, found 838.4021.

## 4. Conclusions

In conclusion, we have developed a fluorescent chemosensor **Rh-C** based on a rhodamine B and coumarin backbone. This probe allowed the selective detection Cu^2+^ in different solvents (THF, MeOH and MeCN) by the naked eye, especially in THF. Additionally, **Rh-C** could also sense Fe^3+^ in MeOH and Cr^3+^ in MeCN by ratiometric fluorescence change *via* a FRET process. Hence, we realize the optical detection of three kinds of common heavy metal ions with a single probe molecule, which might prove beneficial for the strategic design of multifunctional optical sensors.

## Supplementary Materials

Supplementary materials can be accessed at: http://www.mdpi.com/1424-8220/15/1/49/s1.
