# Supplementary material for: One Single Molecule as a Multifunctional Fluorescent Probe for Ratiometric Sensing of Fe3+, Cr3+ and Colorimetric Sensing of Cu2+
Source: Sensors (Basel). 2014 Dec 23;15(1):49–58. doi: 10.3390/s150100049 (PMC4327006; doi:10.3390/s150100049)
Supplement: Supplementary file 1 — Supplementary materials can be accessed at: http://www.mdpi.com/1424-8220/15/1/49/s1. [file sensors-15-00049-s001.pdf]

OPEN ACCESS

sensors

ISSN 1424-8220

www.mdpi.com/journal/sensors

Supplementary Information

One Single Molecule as a Multifunctional Fluorescent Probe for Ratiometric Sensing of Fe^{3+}, Cr^{3+} and Colorimetric Sensing of Cu^{2+}. *Sensors* 2015, *15*, 49-58

Yanqiu Yang [1], Kangkang Yu [2], Liang Yang [1], Jun Liu [1], Kun Li [2,*] and Shunzhong Luo [1,*]

1 Institute of Nuclear Physics &Chemistry, China Academy of Engineering Physics (CAEP), Mianyang 621900, Sichuan, China; E-Mails: yan.qiu.yang@163.com (Y.Y.); leonesta@163.com (L.Y.); ljkxch@163.com (J.L.)

2 College of Chemistry, Sichuan University, Chengdu 610064, Sichuan, China; E-Mail: kangkangyu_1991@163.com

* Authors to whom correspondence should be addressed; E-Mails: kli@scu.edu.cn (K.L.); luoshzh@caep.ac.cn (S.L.); Tel./Fax: +86-288-5415-880 (K.L.); +86-816-2484-241 (S.L.).

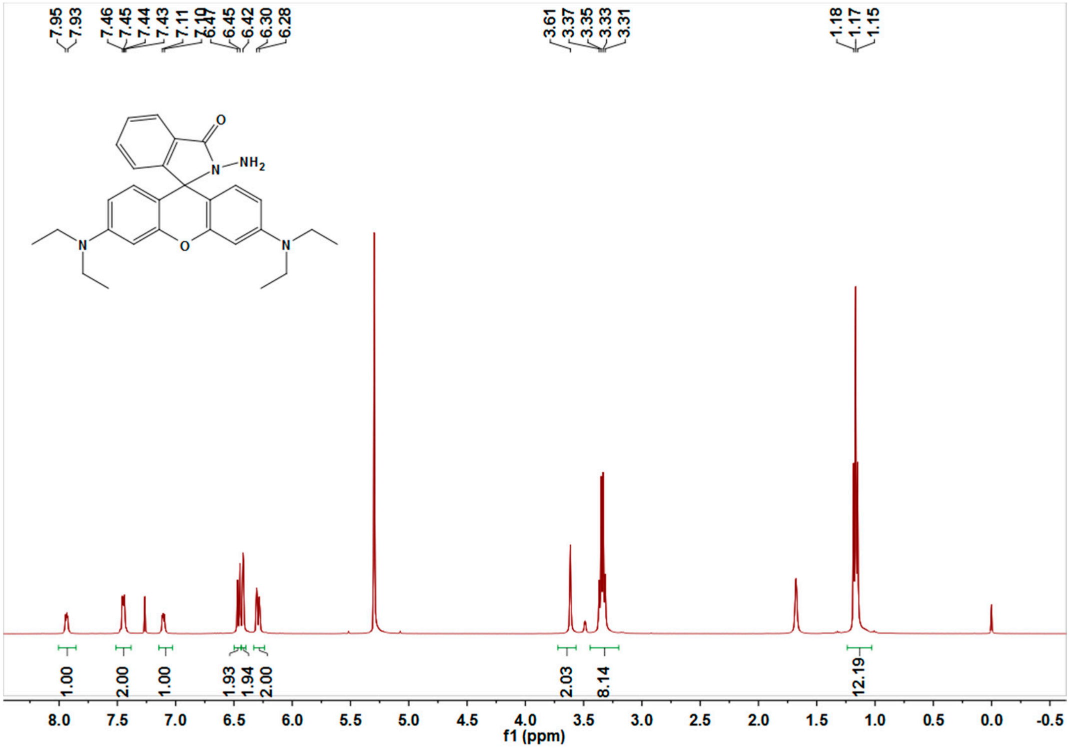

Figure S1. ^{1}H-NMR spectra of **Rh-1** in $CDCl_3$.

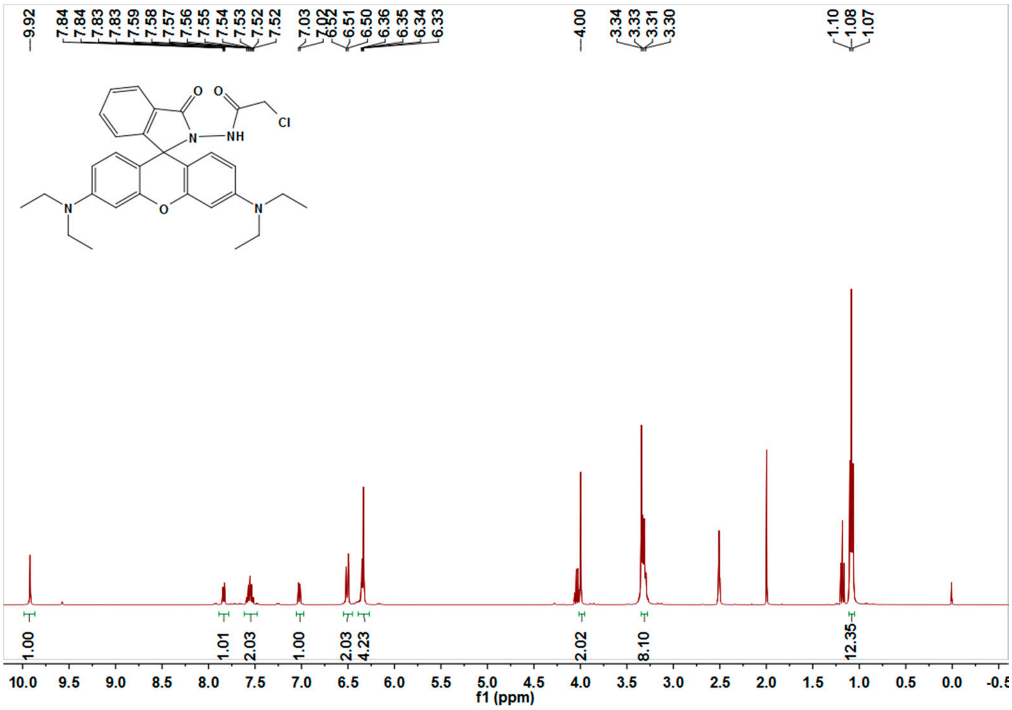

Figure S2. ^{1}H-NMR spectra of **Rh-2** in DMSO-d$_6$.

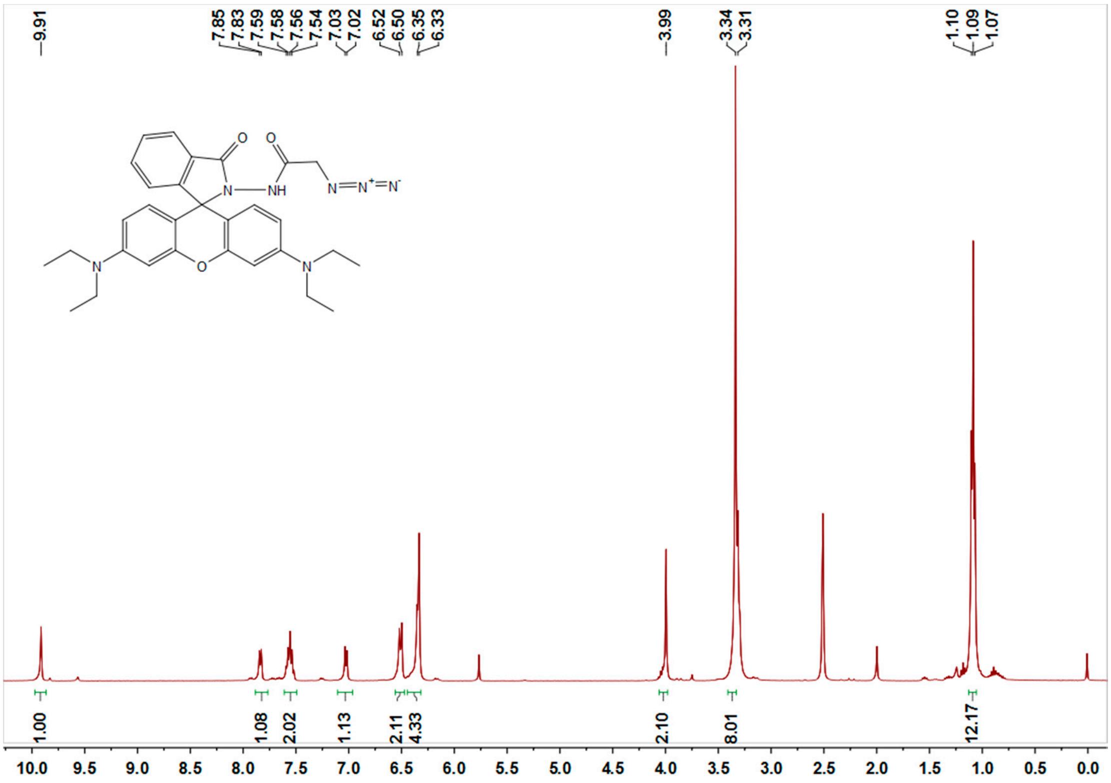

Figure S3. ^{1}H-NMR spectra of **Rh-3** in DMSO-d$_6$.

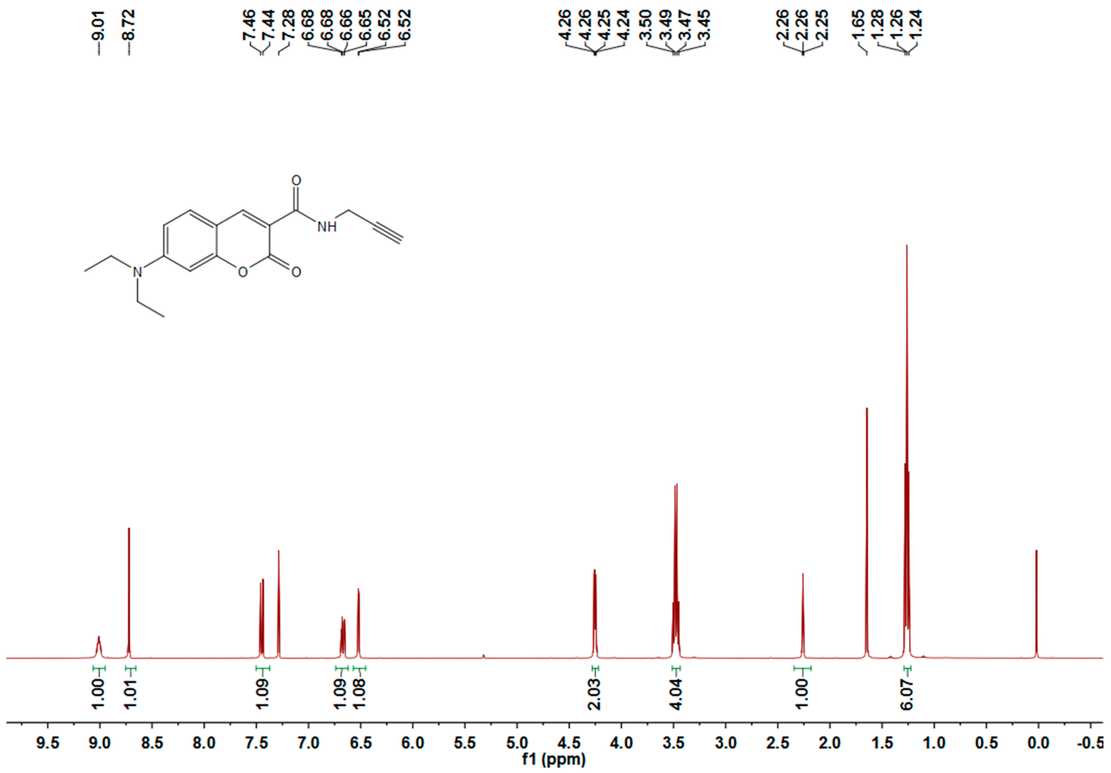

Figure S4. ^{1}H-NMR spectra of **C-3** in $CDCl_3$.

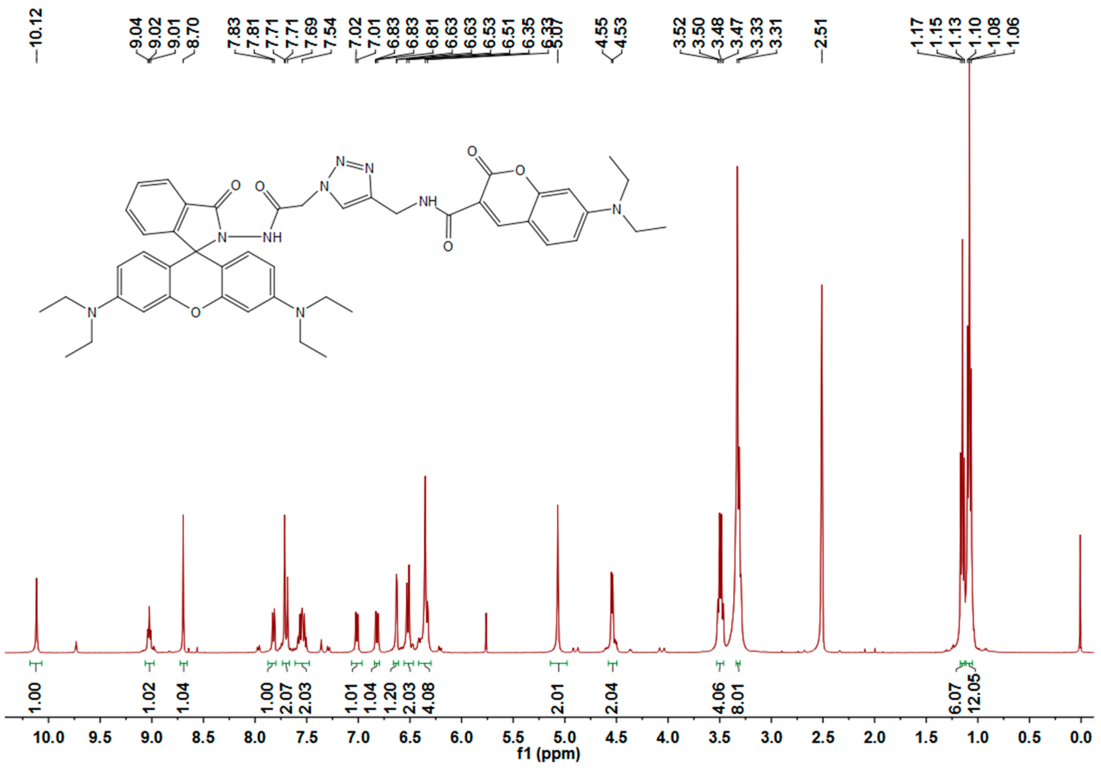

Figure S5. ^{1}H-NMR spectra of **Rh-C** in DMSO-d_6.

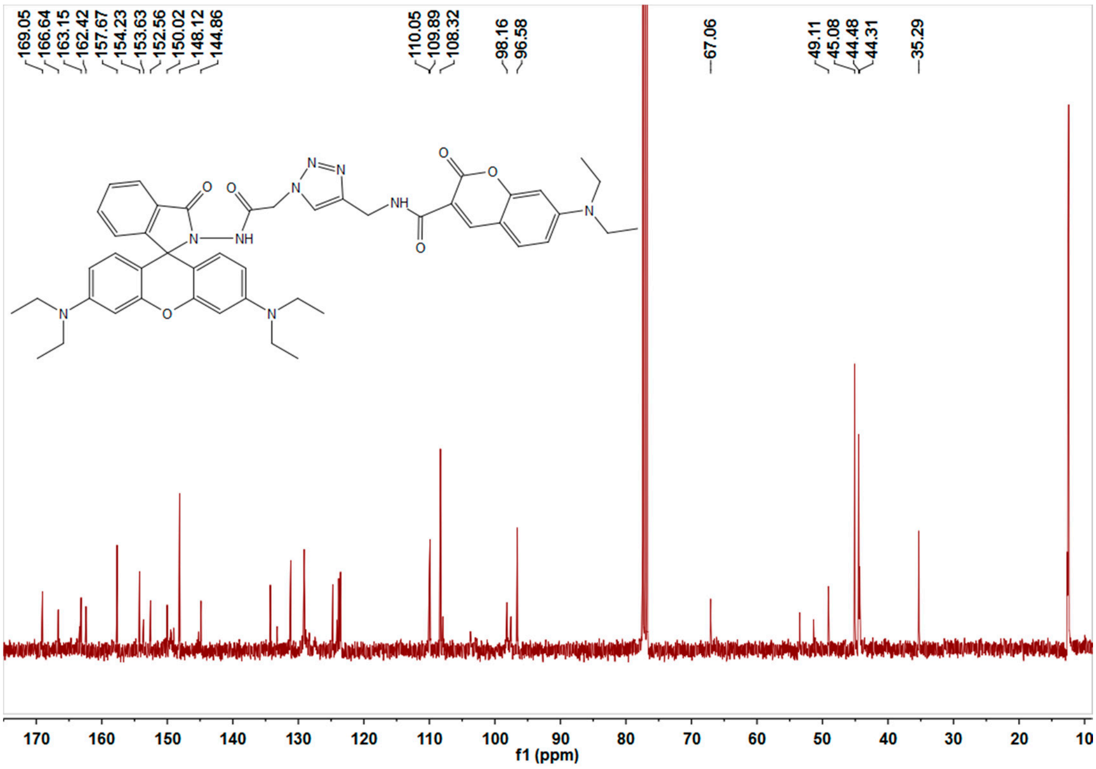

Figure S6. ^{13}C-NMR spectra of **Rh-C** in CD_3Cl.

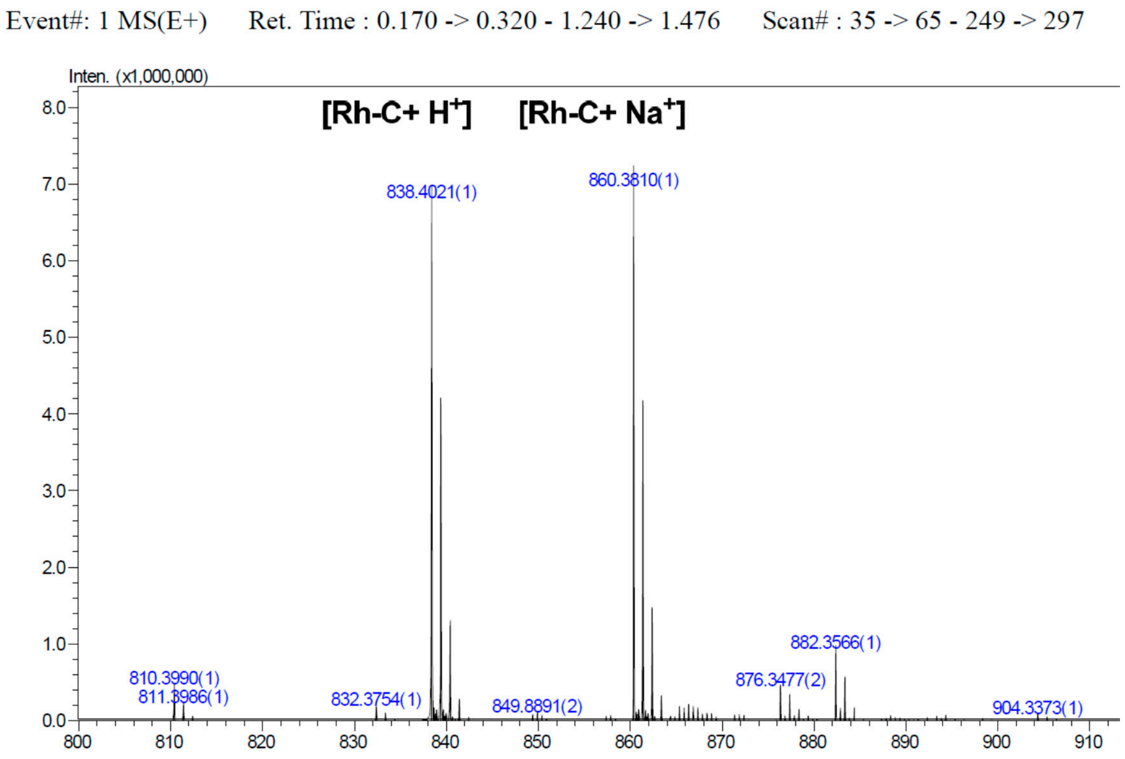

Figure S7. ESI-MS of **Rh-C**.

© 2014 by the authors; licensee MDPI, Basel, Switzerland. This article is an open access article distributed under the terms and conditions of the Creative Commons Attribution license (http://creativecommons.org/licenses/by/4.0/).
